# Proposed protocol for utilising high-flow nasal oxygen therapy in treatment of dogs hospitalised due to pneumonia

**DOI:** 10.1186/s12917-023-03737-7

**Published:** 2023-09-21

**Authors:** Anna-Maija Teppo, Heini Rossi, Minna M. Rajamäki, Heli K. Hyytiäinen

**Affiliations:** Department of Equine and Small Animal Medicine, Faculty of Veterinary Medicine, University of Helsinki, Helsinki, Finland

**Keywords:** Dog, High-flow nasal cannula, Hypoxemia, Non-invasive ventilation, Optiflow, Oxygen supplementation, Respiratory distress

## Abstract

**Background:**

High-flow nasal oxygen (HFNO) therapy is a non-invasive respiratory support method that provides oxygen-enriched, warmed, and humidified air to respiratory-compromised patients. It is widely used in human medical care, but in veterinary medicine it is still a relatively new method. No practical guidelines exist for its use in canine pneumonia patients, although they could potentially benefit from HFNO therapy. This study aims to provide a new, safe, non-invasive, and effective treatment protocol for oxygen supplementation of non-sedated dogs with pneumonia.

**Methods:**

Twenty privately owned dogs with pneumonia will receive HFNO therapy at a flow rate of 1–2 L/kg, and the fraction of inspired oxygen will be determined individually (ranging from 21% to 100%). HFNO therapy will continue as long as oxygen support is needed based on clinical evaluation. Patients will be assessed thrice daily during their hospitalisation, with measured primary outcomes including partial pressure of oxygen, oxygen saturation, respiratory rate and type, days in hospital, and survival to discharge.

**Discussion:**

The proposed protocol aims to provide a practical guideline for applying HFNO to dogs hospitalised due to pneumonia. The protocol could enable more efficient and well-tolerated oxygenation than traditional methods, thus hastening recovery and improving survival of pneumonia patients.

## Background

High-flow nasal oxygen (HFNO) therapy is a non-invasive method of respiratory support. It has been used in human medicine for more than twenty years to treat neonatal, paediatric, and adult patients [1]. Recently, it has gained popularity also in veterinary medicine as an alternative method of oxygen supplementation for animals requiring respiratory support beyond conventional oxygen therapy [2, 3]. However, clinical experience in using HFNO in animals is limited.

The HFNO device warms and humidifies a mixture of oxygen and air (room air or compressed medical air). Humidification is more effective than when the conventional bubble humidifier is used [4], and therefore, patients tolerate higher flows better [3, 5, 6]. This enables more efficient oxygen supply and results in several positive physiological effects in addition to good humidification: reduction of anatomical dead space, a positive end-expiratory pressure effect, and a constant fraction of inspired oxygen (FiO_2_) [5].

Most of the dogs hospitalised due to pneumonia are respiratory-compromised and require additional oxygen at least at the beginning of hospitalisation [3, 7]. Traditionally, supplemental oxygen has been provided via flow-by, nasal prongs/cannulae, masks, collars, hoods, and in severe cases an oxygen cage [3]. Failure of these methods to adequately oxygenate the animal leaves two alternatives: mechanical ventilation or humane euthanasia. However, mechanical ventilation therapy is often not a feasible option. In human medicine, HFNO has proved to be an effective treatment alternative for patients for whom conventional methods of providing oxygen are insufficient but who do not yet need to be intubated [5, 8, 9]. In humans, conventional oxygen therapy can be considered to fail if the target oxygen saturation (SpO_2_) of 90–96% cannot be maintained despite oxygen supply [10], whereas in dogs the threshold values for failure have been reported as SpO_2_ 92–96% and PaO_2_ 70–75 mmHg [11–13]. In humans, compared with conventional oxygen, HFNO has been shown to decrease mortality at 90 days and increase ventilator-free days [14], reduce time in the intensive care unit [15], decrease respiration rate and effort, and increase oxygenation and comfort [16]. Therefore, HFNO can be considered superior to conventional oxygen delivery methods when treating pneumonia patients, including COVID-19 patients [9, 17].

In animals, there is limited experience in using HFNO in clinical practice for treating respiratory diseases, and only a few scientific reports are available [2, 3, 11–13, 18–24]. Moreover, HFNO has been studied in both sedated and non-sedated dogs, healthy and respiratory-compromised dogs, and brachycephalic dogs recovering from anaesthesia. In all of these studies, HFNO has been shown to be safe to use, with minimal or no complications emerging. Patients’ respiratory rate and effort have decreased, and markers of oxygenation improved [11–13, 18, 19, 21, 22]. Despite the promising experiences and reports of using HFNO in respiratory-compromised dogs, no research is available for its use in non-sedated dogs with pneumonia.

## Methods

The aim of this study is to provide a new, safe, non-invasive, readily available, and effective treatment protocol for oxygen supplementation of non-sedated dogs with pneumonia.

### Study design

**Fig. 1:**
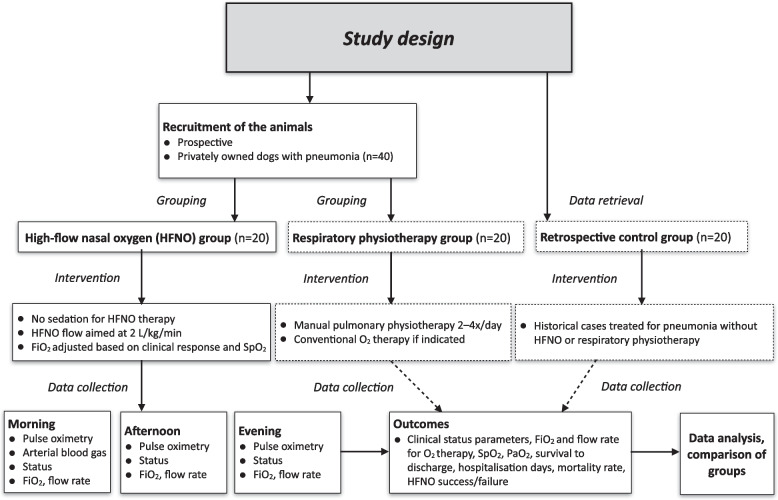
A flow chart of the study design. Abbreviations: FiO_2_=fraction of inspired oxygen, HFNO=high-flow nasal oxygen, PaO_2_=partial pressure of oxygen, SpO_2_=oxygen saturation

The study design and main outcomes are presented schematically in Fig. 1. This study is a randomised parallel group exploratory study, and the current report is concentrated on the description of the HFNO protocol. The recruitment of the participants is performed by the researchers. The personnel (veterinarians, nurses) of the research premise will participate in the implementation of the study plan and collecting the data as the study is carried out simultaneously with the routine hospital treatment of the animals based on their clinical needs. Due to clinical nature of it, blinding is not applicable for this study.

### Animals

Forty privately owned dogs with a diagnosis of pneumonia (either bacterial or aspiration pneumonia) will be prospectively recruited to the study. All patients enrolled in the study will be treated and monitored in the Intensive Care Unit of the University of Helsinki Veterinary Teaching Hospital. Inclusion criteria for enrolment will be PaO_2_ less than 80 mmHg and findings in clinical examinations and radiographs compatible with pneumonia [7, 25, 26]. Dogs with concurrent disease that causes respiratory distress (i.e. cardiac or non-cardiac oedema, trauma), brachycephalic dogs, and dogs weighing less than 3 kg or who are very stressed or anxious while being handled will be excluded. Dog owners will receive verbal and written information on the study details and sign the consent before recruitment. The Finnish Project Authorisation Board has approved the study protocol (ESAVI/37496/2021).

Forty recruited dogs with pneumonia will be randomly assigned (internet-based software generated randomisation, Graphpad.com) into two treatment groups: HFNO and physiotherapy group. Twenty dogs will receive HFNO (HFNO group) instead of conventional oxygen therapy. Otherwise, they will be treated as other pneumonia patients, and they are allowed to receive all other medications needed. The dogs will not be sedated for the study. The dogs in the HFNO group will be compared with another prospective intervention group of 20 dogs receiving respiratory physiotherapy. Dogs in the physiotherapy group will receive conventional oxygen therapy and pulmonary physiotherapy 2–4 times per day. Therapy includes percussion of the chest wall to loosen secretions off the airway walls, manual chest wall vibrations to promote evacuation of the secretions, and postural drainage to further aid the removal of secretions. If the clinical condition of a dog assigned to physiotherapy group does not improve or declines during the study period in a way that more intensive oxygen therapy such as HFNO or ventilation is required, then the required treatments are provided but the dog is excluded from the study. The physiotherapy group was chosen for a comparison group as physiotherapy also aims to improve the oxygenation of the animal with non-invasive methods. Results of the physiotherapy interventions will be published elsewhere. Additionally, both study groups will be compared with a retrospective control group of 20 dogs. The retrospective control group consists of dogs treated for pneumonia in the same facility without receiving physiotherapy or HFNO but with conventional oxygen supply allowed. Retrospective data will be used for controls as any prospective patient fulfilling the inclusion criteria cannot be denied HFNO or physiotherapy due to ethical reasons.

Sample size for this study was calculated based on the assumed difference in the respiratory rate between the experimental arms and the retrospective control group. Estimated standard deviation was 30 for control group and 41 for experimental arms, and estimated detectable difference between the groups was 33. With power of 80% and significance level set at 0.05, the estimated sample size was 20 animals per group. The justification for the estimations was a previous HFNO publication on dogs [12].

### Data collection and outcomes

Data are collected after initiation of HFNO therapy every morning before and 15 min after disconnecting the dog from HFNO, as well as in the afternoon and in the evening while the dog is on HFNO therapy. The following variables are recorded each time: FiO_2_, flow rate, SpO_2,_ body temperature, heart and respiratory rate, respiratory sounds (normal, pronounced, crackles, wheezes) and type (normal, tachypnoea, respiratory distress), nasal/oral discharge, cough, colour of mucous membranes, and capillary refill time. Heart rate and SpO_2_ will be measured with pulse oximetry in three separate consecutive measurements. The pulse oximeter sensor is placed on the dog’s lip or on an abdominal or inguinal skinfold. Heart rate will also be determined by auscultation. Respiratory rate will be assessed by visualising the number of breaths over 1 min. Patient identification and signalment, body weight, and nasal prong interface type (adult vs. paediatric) are documented.

Arterial blood sample will be obtained anaerobically in a syringe (Pico 70, Radiometer, Bronshoj, Denmark) from the dog’s metatarsal artery before initiating HFNO therapy and thereafter every morning as long as the therapy continues. The dogs will go without an oxygen supply for 15 min before the arterial blood sample is collected. Immediately after sample collection, PaO_2_, SpO_2_, and alveolar-arterial gradient will be measured with a blood gas analyser (ABL90 Flex, Radiometer). Oxygen saturation is also measured with a pulse oximeter (Nonin 2500 AV, Jorgen Kruuse, Langeskov, Denmark) several times a day while the dog is on HFNO. The measurements are obtained before starting HFNO therapy, twice every morning, and once in the afternoon and in the evening. The first morning measurement is obtained while the dog is still receiving HFNO. The second measurement is obtained simultaneously with the daily arterial sample once the dog is disconnected from HFNO.

Moreover, to complete the data collection, survival to discharge, hospitalisation days, mortality rate, and HFNO success/failure are recorded. Survival to discharge will be documented as yes or no. Reason for non-survival will be documented as another disease unrelated to lungs, a disease related to lungs, financial, both lungs and financial, or unknown.

All data collected will be stored in the University of Helsinki Veterinary Teaching Hospital’s patient database (Provet Net, Finnish Net Solutions Oy, Finland). In addition, data relevant to the study will be recorded to data sheets and saved to a storage managed and protected by the University of Helsinki. Data will be handled only by the assigned care and research teams.

### High-flow nasal oxygen equipment

**Fig. 2:**
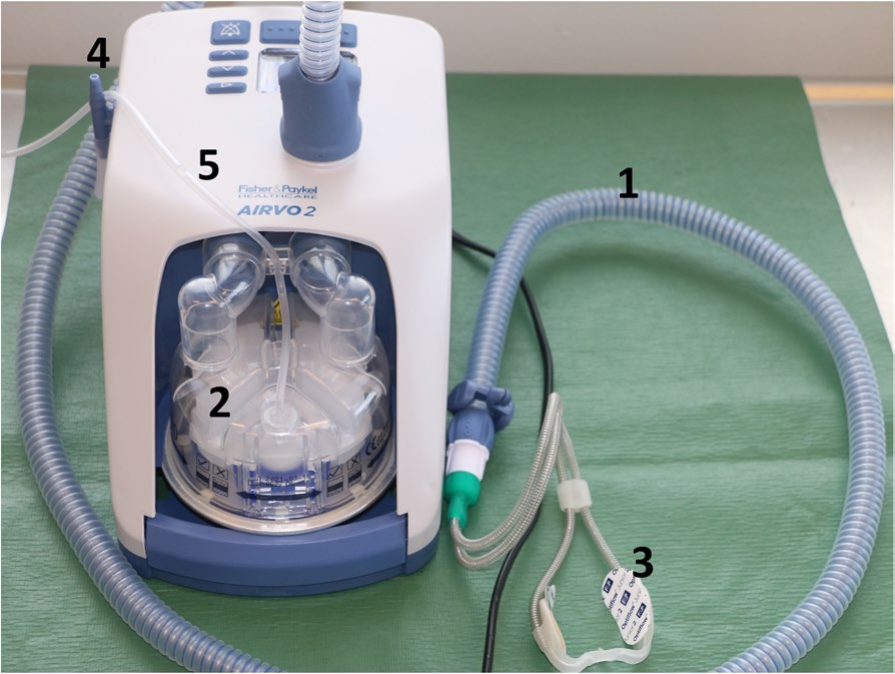
Optiflow Airvo 2 high-flow nasal oxygen device: (1) wire-heated circuit tubing; (2) hot plate-heated humidification chamber system; (3) nasal cannula; (4) oxygen inlet port; (5) sterile water supply

**Fig. 3:**
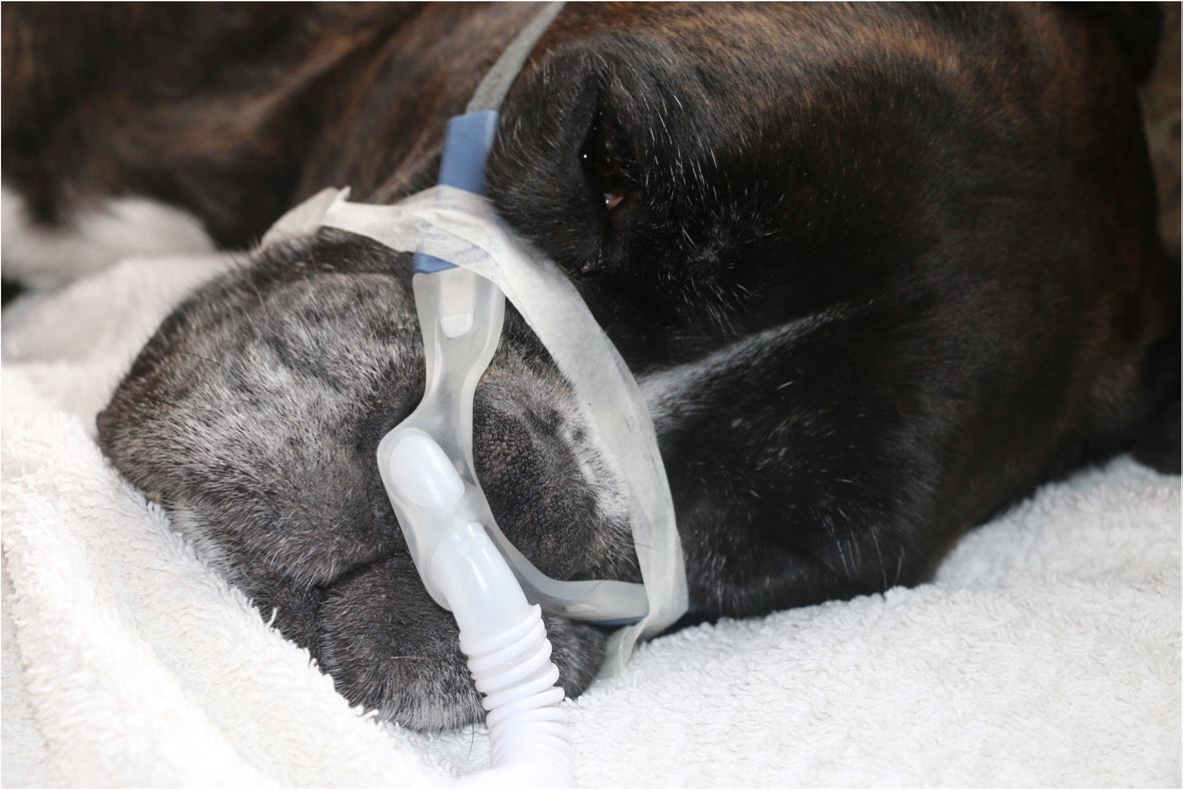
Human adult high-flow nasal oxygen cannula used to deliver air to medium or giant-sized dogs

Optiflow Airvo 2 device (Fig. 2) (Fisher & Paykel Healthcare, Auckland, New Zealand) will be used to deliver high-flow oxygen. The equipment comprises an Airvo 2 high-flow nasal system, an AirSpiral heated breathing tube, a chamber kit, and different sizes of Optiflow interfaces. Optiflow interfaces are the nasal cannula sections of the equipment, which are placed into the dog’s nostrils. They consist of bilateral nasal prongs constructed from soft silicone. The interface includes tubing that connects to the inspiratory circuit either to the side of the face for adult-sized interfaces or around the head for junior-sized interfaces. Unlike conventional nasal oxygen prongs, they do not go deep into the nasal cavity. There are two paediatric, four junior, and three adult interfaces. An individual interface is chosen according to the dog’s size and nasal structure (Fig. 3). The interfaces have been constructed for humans, and paediatric, junior, or small adult interfaces are likely to fit best for most dogs. The interface will be selected such that they will not occlude more than 50% of the patient's nares based on manufacturer’s and standard human recommendations. They will be fixed with a rubber band tied behind the dog’s ears. Tapes or 2–3 simple interrupting sutures can be used if additional support is needed (Fig. 3).

The Optiflow Airvo 2 device blends high flow rates of oxygen and air (room air or compressed medical air) and warms and humidifies the gas mixture. Hot plate-heated humidification chamber system is used to warm the gas mixture to 37℃ (but also 31℃ and 34℃ could be used) and humidify it to 100% relative humidity before administering it to the patient with wire-heated tubing. Adult-sized circuits can provide flow rates of 60 L/min and paediatric ones 25 L/min. The FiO_2_ could be preselected to range from 21% to 100%. Flow rate and temperature are chosen from the device. The amount of oxygen is chosen from a conventional oxygen wall supply device to which the Optiflow Airvo 2 is connected.

### Therapy protocol

In this study, an HFNO flow rate of 1 L/kg/min with 30–40% oxygen fraction and a temperature of 37℃ will be used for the first 30 min. If no adverse effects are observed, the flow rate will be increased to 1.5 L/kg/min for 15 min and then to 2 L/kg/min. The increases are implemented to be within the dog’s tolerance and only until sufficient oxygenation is achieved. During the first 1–2 hours up to 100% oxygen can be used if necessary. Flow rates will be reduced when the patient’s respiratory function improves or if there are concerns with tolerance. The veterinarian treating the dog will determine the flow rate and FiO_2_. If the dog does not tolerate HFNO therapy, it will be excluded from the study and conventional oxygen therapy initiated instead.

Once oxygen therapy is no longer clinically needed, patients will be weaned off the HFNO gradually. The inspired oxygen fraction will be decreased before the flow rate. FiO_2_ will be decreased gradually over 0.5–3 hours until being approximately 21%. Once FiO_2_ of 21% is reached, the HFNO flow rate will be decreased gradually 10–25% every 5–10 min. Once the animal is sufficiently active to no longer tolerate HFNO, the therapy will be discontinued.

## Discussion

This study aims to provide a new, safe, non-invasive, readily available, and effective treatment protocol for oxygen supplementation of non-sedated dogs with pneumonia. If the protocol proves successful, it could be a new practical guideline for applying HFNO to this patient group. The proposed protocol can enable more efficient and well-tolerated oxygenation than traditional methods, thus hastening recovery and improving survival and well-being of pneumonia patients. The equipment needed is relatively inexpensive, easy to use, and therefore available to most clinicians. The proposed protocol and new knowledge gained in this study will make it easier to adopt HFNO for clinical use.

This will be the first study in which HFNO is used solely on canine pneumonia patients. Patients in previously published HFNO-related studies on dogs have been either healthy [19–21] or respiratory-compromised [11–13, 18, 22] for various reasons. Some patients have had pneumonia, most of them because of aspiration, but other diseases, such as cardiac or non-cardiac oedema, or trauma have also been common reasons for needing respiratory support. In previously published studies [11–13, 18–22], most dogs have been at least lightly sedated to accept the nasal prongs, noise, and feeling of airflow caused by the HFNO device. In this study, HFNO will be used without sedation. By not sedating the dogs, patient safety will increase, and less monitoring work will be required. However, it is possible that some dogs will not accept the nasal prongs or will move around too much since they are not sedated, thus complicating the use of HFNO.

In human medicine, patients’ experience of HFNO is well established. Human patients usually find facial interface relatively comfortable [27, 28]. However, also negative experiences have been reported. Patients have complained of nasal irritation, alterations in sense of smell after treatment, and displacement of nasal prongs [6, 29]. Moreover, the noise from the device during HFNO treatment disturbs some patients [27]. Patients may also find the warmth of the inspired air uncomfortable [6, 30].

In dogs, especially displacement of the nasal prong could be a problem since the facial interface is constructed for human facial structures and most dogs’ anatomy is obviously different. Even though there are different sizes of nasal prongs available they may not fit properly into the dogs’ nose [3]. It is also possible that dogs, as well as some humans, find the warm air and noise produced by HFNO uncomfortable. Patient mobility is also reduced relative to conventional nasal oxygen since the air tube of the HFNO device is only 1.75 m long. However, none of these have been reported to be a problem and most non-sedated dogs have tolerated HFNO well [13, 18, 21].

Only a few complications related to the use of HFNO have been reported previously. In three studies [18, 21, 22], some dogs demonstrated a significant increase in partial pressure of carbon dioxide possibly because of sedation. Ingestion of air was noted in three studies [19, 21, 22], but only one case was severe enough that a gastroesophageal tube needed to be placed to resolve the situation [22]. In one study [13] one dog developed pneumothorax, and in another study [18] one dog had pre-existing pneumothorax. Both resolved when HFNO therapy was discontinued. The complications were considered minor, easily resolved, and the overall experiences of HFNO were encouraging [3, 11–13, 18–22].

In conclusion, the proposed methodology is expected to provide improved oxygenation, decreased respiratory effort, and enhanced survival relative to traditional methods. It could also shorten hospitalisation time, improving dogs’ well-being and reducing owners’ expenses. This may lead to better owner compliance also in severe pneumonia cases, which in turn means more patients recovering and surviving to discharge.

## Data Availability

Data sharing is not applicable as no datasets were generated or analysed during the study.

## Abbreviations

FiO_2_: Fraction of inspired oxygen
HFNO: High-flow nasal oxygen
PaO_2_: Partial pressure of oxygen
SpO_2_: Oxygen saturation

## References

[1] Ward JJ (2013). High-flow oxygen administration by nasal cannula for adult and perinatal patients. Respir Care..

[2] Krawec P, Marshall K, Odunayo A (2022). A Review of High Flow Nasal Cannula Oxygen Therapy in Human and Veterinary Medicine. Top Companion Anim Med..

[3] Ramesh M, Thomovsky E, Johnson P (2021). Conventional versus high-flow oxygen therapy in dogs with lower airway injury. Can J Vet Res..

[4] Chanques G, Constantin J, Sauter M, Jung B, Sebbane M, Verzilli D (2009). Discomfort associated with underhumidified high-flow oxygen therapy in critically ill patients. Intensive Care Med..

[5] Nishimura M (2016). High-Flow Nasal Cannula Oxygen Therapy in Adults: Physiological Benefits, Indication, Clinical Benefits, and Adverse Effects. Respir Care..

[6] Roca O, Riera J, Torres F, Masclans JR (2015). High-flow oxygen therapy in acute respiratory failure. Respir Care..

[7] Dear JD (2020). Bacterial Pneumonia in Dogs and Cats: An Update. Vet Clin North Am Small Anim Pract..

[8] Maury E, Alves M, Bigé N (2016). High-flow nasal cannula oxygen therapy: more than a higher amount of oxygen delivery. J Thorac Dis..

[9] Oczkowski S, Ergan B, Bos L, Chatwin M, Ferrer M, Gregoretti C (2022). ERS clinical practice guidelines: high-flow nasal cannula in acute respiratory failure. Eur Respir J..

[10] Siemieniuk RA, Chu DK, Kim LH, Güell-Rous MR, Alhazzani W, Soccal PM (2018). Oxygen therapy for acutely ill medical patients: a clinical practice guideline. BMJ..

[11] Jagodich TA, Bersenas AME, Bateman SW, Kerr CL (2020). High-flow nasal cannula oxygen therapy in acute hypoxemic respiratory failure in 22 dogs requiring oxygen support escalation. J Vet Emerg Crit Care..

[12] Pouzot-Nevoret C, Hocine L, Nègre J, Goy-Thollot I, Barthélemy A, Boselli E (2019). Prospective pilot study for evaluation of high-flow oxygen therapy in dyspnoeic dogs: the HOT-DOG study. J Small Anim Pract..

[13] Frischer R, Daly J, Haggerty J, Guenther C (2022). High-flow nasal cannula improves hypoxemia in dogs failing conventional oxygen therapy. J Am Vet Med Assoc..

[14] Frat JP, Thille AW, Mercat A, Girault C, Ragot S, Perbet S (2015). High-Flow Oxygen through Nasal Cannula in Acute Hypoxemic Respiratory Failure. N Engl J Med..

[15] Teng XB, Shen Y, Han MF, Yang G, Zha L, Shi JF (2021). The value of high-flow nasal cannula oxygen therapy in treating novel coronavirus pneumonia. Eur J Clin Invest..

[16] Hyzy RC. Heated and humidified high-flow nasal oxygen in adults: Practical considerations and potential applications. Waltham (MA): UpToDate; 2020 (cited 2023 Feb 9). Available from: https://www.uptodate.com/contents/heated-and-humidified-high-flow-nasal-oxygen-in-adults-practical-considerations-and-potential-applications.

[17] Jiang B, Wei H (2020). Oxygen therapy strategies and techniques to treat hypoxia in COVID-19 patients. Eur Rev Med Pharmacol Sci..

[18] Keir I, Daly J, Haggerty J, Guenther C (2016). Retrospective evaluation of the effect of high flow oxygen therapy delivered by nasal cannula on PaO2 in dogs with moderate-to-severe hypoxemia. J Vet Emerg Crit Care (San Antonio)..

[19] Daly JL, Guenther CL, Haggerty JM, Keir I (2017). Evaluation of oxygen administration with a high-flow nasal cannula to clinically normal dogs. Am J Vet Res..

[20] Harduin C, Allaouchiche B, Nègre J, Goy-Thollot I, Barthélemy A, Fougeray A (2021). Impact of flow and temperature on non-dyspnoeic dogs' tolerance undergoing high-flow oxygen therapy. J Small Anim Pract..

[21] Jagodich TA, Bersenas AME, Bateman SW, Kerr CL (2019). Comparison of high flow nasal cannula oxygen administration to traditional nasal cannula oxygen therapy in healthy dogs. J Vet Emerg Crit Care (San Antonio)..

[22] Jagodich TA, Bersenas AME, Bateman SW, Kerr CL (2020). Preliminary evaluation of the use of high-flow nasal cannula oxygen therapy during recovery from general anesthesia in dogs with obstructive upper airway breathing. J Vet Emerg Crit Care (San Antonio)..

[23] Floyd E, Danks S, Comyn I, Mackenzie C, Marr CM (2022). Nasal high flow oxygen therapy in hospitalised neonatal foals. Equine Vet J..

[24] Whitney J, Keir I (2023). Clinical review of high-flow nasal oxygen therapy in human and veterinary patients. Front Vet Sci..

[25] Hawkins EC, Nelson RW, Couto CG (2020). Disorders of the Pulmonary Parenchyma and Vasculature. Small Animal Internal Medicine.

[26] Viitanen SJ, Lappalainen A, Rajamäki MM (2015). Co-infections with respiratory viruses in dogs with bacterial pneumonia. J Vet Intern Med..

[27] Lenglet H, Sztrymf B, Leroy C, Brun P, Dreyfuss D, Ricard JD (2012). Humidified high flow nasal oxygen during respiratory failure in the emergency department: Feasibility and efficacy. Respir Care..

[28] Lee CC, Mankodi D, Shaharyar S, Ravindranathan S, Danckers M, Herscovici P (2016). High flow nasal cannula versus conventional oxygen therapy and non-invasive ventilation in adults with acute hypoxemic respiratory failure: A systematic review. Respir Med..

[29] Renda T, Corrado A, Iskandar G, Pelaia G, Abdalla K, Navalesi P (2018). High-flow nasal oxygen therapy in intensive care and anaesthesia. Br J Anaesth..

[30] Mauri T, Galazzi A, Binda F, Masciopinto L, Corcione N, Carlesso E (2018). Impact of flow and temperature on patient comfort during respiratory support by high-flow nasal cannula. Crit Care..

